# Skin cancer screening behaviours among individuals with a strong family history of malignant melanoma

**DOI:** 10.1038/sj.bjc.6605942

**Published:** 2010-10-26

**Authors:** N A Kasparian, J K McLoone, B Meiser, P N Butow, J M Simpson, G J Mann

**Affiliations:** 1 School of Women's and Children's Health, Faculty of Medicine, University of New South Wales, Kensington, NSW 2052, Australia; 2 Psychosocial Research Group, Department of Medical Oncology, Prince of Wales Hospital, Randwick, NSW 2031, Australia; 3 Prince of Wales Clinical School, Faculty of Medicine, University of New South Wales, Kensington, NSW 2052, Australia; 4 Centre for Medical Psychology and Evidence-Based Decision-Making, School of Psychology, University of Sydney, Sydney, NSW 2006, Australia; 5 School of Public Health, University of Sydney, Sydney, NSW 2006, Australia; 6 Westmead Institute for Cancer Research, University of Sydney at Westmead Millennium Institute and Melanoma Institute Australia, Westmead, NSW 2145, Australia

**Keywords:** melanoma, family history, genetics, skin self-examination, clinical skin examination, anxiety

## Abstract

**Background:**

This study examined the prevalence and correlates of skin cancer screening behaviours among individuals at high risk of developing melanoma due to strong family history.

**Methods:**

A total of 120 individuals with a known family-specific *CDKN2A* mutation (72% response rate) completed a self-report questionnaire assessing annual frequency of skin self-examination (SSE), clinical skin examination (CSE) and a variety of potential demographic, clinical and psychosocial correlates.

**Results:**

In the past 12 months, 50% of participants reported engaging in SSE at least four times, and 43% of participants had undergone at least one CSE. Engagement in SSE was associated with doctor recommendation (*β*=1.77, *P*=0.001), confidence in one's ability to perform SSE (*β*=1.44, *P*<0.0001), positive beliefs about melanoma treatment (*β*=0.77, *P*=0.002) and intention to perform SSE in the future (*β*=1.69, *P*<0.0001). These variables accounted for 59% of the variance in SSE behaviour. Further, information-seeking style moderated the relationship between anxiety and SSE (*β*=1.02, *P*=0.004). Annual uptake of CSE was associated with doctor recommendation (*β*=2.21, *P*<0.0001) and intention to undergo CSE in the future (*β*=1.19, *P*=0.001).

**Conclusion:**

In comparison with clinical guidelines, it appears that individuals at high risk of developing melanoma engage in suboptimal levels of skin surveillance. Improved doctor–patient communication, as well as psycho-education and behavioural support, may be viable means of improving early skin cancer detection behaviours in this high-risk population.

Despite more than 30 years of community education on skin cancer prevention and early detection, Australia retains the unwanted reputation as the melanoma epicentre of the world. With over 10 000 cases diagnosed annually, melanoma is the fourth most common cancer in Australia, with one in 14 males and one in 23 females expected to develop melanoma in their lifetime (Tracey *et al*, 2007). National melanoma mortality is relatively stable and lower than that for other common cancers; however, melanoma has a disproportionately heavy impact on productive years of life lost because it is the most common cancer in young adults aged between 15 and 45 years (Australian Institute of Health and Welfare Australasian Association of Cancer Registries, 2004). Although the 5-year survival rate for localised melanoma is 96%, 5-year survival rates for regional and distant-stage diseases are 63 and 34%, respectively. With ∼20% of melanomas diagnosed with local or regional spread, the need to identify and address barriers to early disease detection is clear, particularly in those at increased risk.

An estimated 10% of melanoma cases will have at least one (and most only one) first-degree relative (FDR) with a confirmed melanoma diagnosis. Many of these familial clusters will be because of chance or shared environmental influences; however, at a minimum, 2% of all cases represent genuine high-risk kindreds potentially resulting from the inheritance of uncommon, major melanoma susceptibility genes (Easton *et al*, 1991; Aitken *et al*, 1994). Germline mutations in two genes, *CDKN2A* and (rarely) *CDK4*, have been shown to cause inherited melanoma susceptibility with high penetrance (Bishop *et al*, 2002; Hayward, 2003; Kefford and Mann, 2003), and a third such locus has recently been identified on chromosome 1p22 (Gillanders *et al*, 2003). There is wide variation in estimates of risk conferred by carrying a pathogenic *CDKN2A* mutation, and strong evidence suggests that risk varies across different populations (Bishop *et al*, 2002), and is influenced by other independent risk factors for melanoma such as level of exposure to ultraviolet radiation (Cannon-Albright *et al*, 1994; Goldstein *et al*, 1998), skin pigmentation and freckling (Palmer *et al*, 2000; van der Velden *et al*, 2001), and potentially by modifier genes which may, in certain families, also be associated with the presence of atypical nevi (Tucker *et al*, 1993). In the context of familial melanoma in Australia, estimates of risk conferred by *CDKN2A* mutation are 10–20 times greater than that in the general population.

Evidence-based guidelines for the clinical management of individuals at high risk of developing melanoma recommend that such persons be educated to recognise and document lesions suggestive of melanoma, perform skin self-examination (SSE) regularly (i.e., monthly) and engage in bi-annual full body skin examination conducted by a clinician and supported by dermoscopy and total body digital photography, as required (Australian Cancer Network, 2008). While there have been no controlled trials evaluating the impact of clinical skin examination (CSE) on melanoma mortality, prospective studies of high-risk groups have repeatedly demonstrated that the average thickness of melanomas detected is reduced under regular surveillance (e.g., Carli *et al*, 2003). Therefore, to the extent that the prognosis of primary melanoma is related to its thickness, surveillance may be inferred to benefit such patients. It is also well established that a large proportion of melanoma patients (44–71%) detect their own malignant lesion (Brady *et al*, 2000; Robinson *et al*, 2002; McPherson *et al*, 2006). Case–control evidence indicates that performance of SSE is associated with a reduced risk of advanced disease (Berwick *et al*, 1996), and that heightened skin awareness may increase the chances of survival (Berwick *et al*, 2005). Thus, SSE and CSE are critical components of early detection programmes in melanoma.

Yet, despite the importance of skin surveillance, very little is known about the prevalence or predictors of these behaviours among those at highest risk of melanoma. Widely accepted theoretical models of health behaviour, such as the Transactional Model of Stress and Coping, posit that the beliefs and appraisals people form in relation to their health and illness experiences are closely related to the decisions they make and the ways in which they cope with health threats (Folkman and Guer, 2000; Glanz *et al*, 2002). According to the Transactional Model, variations in these appraisals will evoke different individual responses to the same health threat. When faced with a stressor (e.g., melanoma risk), a person evaluates the potential threat and forms a judgment about the personal significance of the event as stressful, controllable, challenging or irrelevant (i.e., perceived risk and causal attributions). Secondary appraisal follows, which is an assessment of the person's coping resources and options, as well as beliefs about what one can do about the situation (i.e., self-efficacy and response efficacy). Actual coping efforts aimed at regulation of the problem give rise to outcomes of the coping process, including emotional well-being and health behaviours. Dispositional coping styles (e.g., information-seeking style) may also have a role, with generalised ways of behaviour influencing a person's emotional or functional response to the stressor, and these styles generally remain relatively stable across time and situations. When considering adherence to melanoma-related health behaviours such as SSE and CSE, according to the Transactional Model, an individual is more likely to engage in these behaviours if he or she perceives the threat of melanoma as high, if one believes one is capable of performing the required actions and that these actions will be effective in reducing melanoma risk, and if the required knowledge and support is available. However, application of these models to the setting of familial melanoma has not been investigated.

Therefore, the aims of the present study were two-fold: (1) to examine the annual frequency of SSE and CSE practices in a sample of individuals at high risk of developing melanoma due to strong family history and an identified family-specific mutation in the *CDKN2A* gene; (2) to identify the demographic, clinical and psychosocial factors associated with the uptake of these skin cancer screening behaviours.

## Materials and methods

### Participants

Individuals with a strong family history of melanoma (i.e., families comprising at least three relatives with a confirmed melanoma diagnosis) and a known family-specific *CDKN2A* mutation were ascertained via the Westmead Institute for Cancer Research/University of Sydney centre of the Genetic Epidemiology of Melanoma study. This is now part of the international GenoMEL consortium (http://www.genomel.org), a multidisciplinary study of the genetic epidemiology of melanoma (Holland *et al*, 1999; Bishop *et al*, 2002; Goldstein *et al*, 2006). A detailed description of ascertainment into the larger study has been published elsewhere (Holland *et al*, 1999). Briefly, multiple-case melanoma families have been ascertained from South Eastern Australia to the Sydney arm of this study for over 18 years through either: (i) a family member who attended the Sydney Melanoma Unit/Melanoma Institute Australia (the largest dedicated melanoma treatment service in the world), the Victorian Melanoma Service or other clinics, for treatment of melanoma, (ii) referral from health professionals such as clinical geneticists or dermatologists or occasionally, (iii) self-referral after media publicity of melanoma. Data on family structure, cancer history, illness characteristics, skin phenotype, other melanoma risk factors and genotype are collected as part of this study; however, participants are not systematically provided with any educational materials about melanoma risk.

Fully consented individuals were eligible for the present study if a family-specific mutation in the *CDKN2A* gene had been identified via the GenoMEL protocol. Ineligibility criteria included: having previously undergone genetic testing for melanoma risk in the clinical setting, inability to give informed consent and current diagnosis with metastatic cancer.

### Procedure

This paper presents the baseline data from a prospective cohort study of candidates for genetic testing for melanoma risk (Kasparian *et al*, 2009b). Identification of potentially pathogenic mutations in 18 families made genetic testing possible and, in accordance with the larger study protocol, all participating members of these families (*N*=176) were informed by letter about the availability of genetic counselling and testing in January 2005 and were simultaneously offered to participate in the current study. Individuals who did not decline study participation were telephoned 14 days after invitation letters were mailed to determine their interest in participating. Interested individuals were mailed the first (‘baseline’) questionnaire and a pre-paid envelope. Reminder letters and phone calls were made as appropriate to participants who did not complete the questionnaire within a specified time. The appropriate Institutional Review Board gave approval and informed consent was obtained from all participants.

### Measures

Clinical characteristics were accessed through the Sydney-based Genetic Epidemiology of Melanoma database and a pedigree was created for each participating family, containing data on: total number of individuals affected by melanoma, total number of FDRs and second-degree relatives (SDRs) deceased due to melanoma, and personal history of melanoma. In the absence of a published algorithm for calculation of lifetime risk of melanoma, estimated risk of being a *CDKN2A* mutation carrier was assigned for each participant before genetic testing. Given the presence of a family-specific *CDKN2A* mutation, participants with a personal history of melanoma were assigned a 100% risk of carrying a mutation. Amongst unaffected participants, those whose closest affected relative was a FDR or SDR were assigned a 50 or 25% risk, respectively, and those with no known FDR or SDR with melanoma were assigned a risk of 12.5%.

The study questionnaire elicited the following data:

1. Demographic characteristics: age, sex, marital status, educational level, country of birth and occupational environment.
2. Perceived risk of developing melanoma was assessed using five items described in an earlier publication (Kasparian *et al*, 2009b). On the basis of factor analytic results, a summary score was calculated for these items for analysis (Cronbach's alpha=0.73).
3. Causal attributions for melanoma were assessed via 11 items based on our previous qualitative work (Kasparian *et al*, 2007, 2008). Three causal factors were assessed: sun exposure (five items), genetics (three items) and uncontrollable factors (e.g., chance, three items). Participants rated the importance of each item as a cause of melanoma on a 5-point Likert rating scale from 1 (‘not at all important’) to 5 (‘extremely important’). A summary score was calculated for each of the three factors for analysis.
4. Self-efficacy for SSE was assessed using a single item to identify participants’ confidence in their ability to accurately and regularly conduct SSE. Response options ranged from 1 (‘not at all confident’) to 5 (‘extremely confident’), with higher scores indicating greater self-efficacy.
5. Response efficacy was assessed using two items to identify participants’ beliefs about the efficacy of: (i) CSE as a means of early melanoma detection and (ii) medical treatment as a means of curing melanoma. Participants responded to each item using a 5-point Likert scale from 1 (‘strongly disagree’) to 5 (‘strongly agree’). Higher scores indicated more negative beliefs about response efficacy.
6. Patient communication with their doctor about family history of melanoma was assessed using one item. Participants were asked whether they had ever discussed their family history of melanoma with their doctor. Response options were ‘yes’, ‘no’ and ‘unsure’.
7. Doctor recommendation was assessed using three items. For SSE, participants were asked to indicate whether a doctor or other health professional had ever: (i) recommended the person to regularly examine his or her skin for signs of changes in spots or moles and (ii) provided instruction or education about the best way to perform this procedure. For CSE, one item assessed whether a doctor had ever suggested the participant see a health professional for skin cancer screening.
8. Behavioural intentions were assessed using two items to identify the likelihood that the participant would engage in SSE or CSE in the next 12 months. Participants indicated their screening intentions on a 5-point Likert scale ranging from 1 (‘not at all likely’) to 5 (‘extremely likely’).
9. Melanoma-specific distress was assessed with the 15-item Impact of Events Scale (IES; Horowitz *et al*, 1979). Participants rated the frequency of intrusive and avoidant cognitions and behaviours regarding their melanoma risk using a 4-point frequency scale. A total score ⩾40 is considered indicative of a significant stress response (Horowitz *et al*, 1979; Cella *et al*, 1990). Internal consistency for the IES total score was 0.89.
10. General anxiety and depression: The Hospital Anxiety and Depression Scale has two 7-item subscales measuring anxiety and depression (Zigmond and Snaith, 1983). Each item has four response options ranging from 0 (‘not at all’) to 3 (‘very much’), yielding scores from 0 to 21 for each subscale. Subscale scores ⩾8 indicate potentially elevated distress (Zigmond and Snaith, 1983). Internal consistency was 0.85 and 0.74 for the anxiety and depression subscales, respectively.
11. Information-seeking style was assessed using the Miller Behavioural Style Scale (Miller, 1987; Muris and Schouten, 1994). Respondents were asked to imagine four hypothetical stress-invoking scenarios of a largely uncontrollable nature. Each scenario was followed by eight responses indicative of either high or low information-seeking (or monitoring) style. For the analysis, individuals were categorised as either low or high monitors on the basis of how they anticipated their response(s) to these threat-related cues, using a median split (Miller, 1996).
12. Screening behaviours: SSE and CSE behaviours were the outcome variables for this study. Skin self-examination was defined as ‘the careful and deliberate checking for changes in spots or moles on all areas of your skin, including those areas rarely exposed to the sun’ (Manne *et al*, 2004). Participants indicated the number of times they had engaged in SSE over the past 12 months using a 5-point Likert scale ranging from 1 (‘not at all’) to 5 (‘weekly’). Participants also indicated their use of a mirror or the help of another person when performing SSE on a 5-point scale from 1 (‘never’) to 5 (‘always’). Participants were then asked to indicate whether they had ever had a CSE and if so, the number of times they had undertaken CSE in the past 12 months, giving the approximate date of each examination (Manne *et al*, 2004).

### Data analysis

Data were analysed using SPSS 17.0 and SAS 9.1. Differences between participants and non-participants for non-psychological variables were assessed using Pearson's *χ*^2^-tests, linear-by-linear association tests or *t*-tests, as appropriate. For the bivariate analyses, the behavioural outcome variables were treated as ordinal variables. Accordingly, Mann–Whitney *U*-tests or Kruskal–Wallis tests were used to examine associations between categorical predictor variables and behavioural outcomes, and Spearman's rank correlation coefficients (*r*_s_) were used to examine associations between continuous predictor variables and behavioural outcomes.

To assess determinants of screening behaviours, two separate linear regressions were performed. Predictor variables with *P*⩽0.10 in the bivariate analyses were included in initial multivariate models (Hosmer and Lemeshow, 2000). A progressive, backward elimination modelling strategy was employed until a final model was obtained containing only variables with *P*<0.05. Owing to their importance, age, sex, educational level and marital status were included in all regression models as potential confounders. When the preliminary final model for each regression analysis had been built, each potential confounder was removed, one at a time. If its removal resulted in a >10% change in the coefficient of any predictor, the variable was considered a confounder and was retained in the final model (Kleinbaum *et al*, 1987). Multi-collinearity of predictor variables was checked but no problem was detected. Two-way interactions among terms in the final models were tested by adding them one at a time. In all regression models, analyses included data from members of the same family. To account for potential dependence among these data, correlations among responses of individuals in the same family cluster were allowed for using generalised estimating equations for linear regression (Liang and Zeger, 1986). For scale variables, the reported value of *β* represents the change in the outcome variable per unit increase in the scale.

## Results

### Response rates and sample characteristics

Of the 176 individuals who were considered eligible for study participation, contact details were not available for 10, therefore, 166 individuals were approached for participation. Of these, 120 individuals returned questionnaire data, yielding a response rate of 72% among eligible, successfully contacted participants. The mean age of the sample was 50 years (s.d.=15.5), with males (48%) and females (52%) represented equally. The majority of the sample (74%) was married, 11% had completed a university degree and 96% had been born in Australia. In terms of occupational environment, 52% of participants worked primarily indoors, 22% worked outdoors and 23% spent equal amounts of time indoors and outdoors. One-third of the sample (31%) had a personal history of melanoma, 62% had at least three FDRs or SDRs with a previous melanoma and 41% had lost a family member due to melanoma. Participants and non-participants did not differ significantly by age, sex, personal history of melanoma or number of affected relatives. Psychological characteristics of the sample are shown in Table 1. The majority of the sample (92% of affected and 69% of unaffected participants) reported having discussed their family history of melanoma with their doctor. Participants without a personal history of melanoma were less likely to have discussed their family history with their doctor than those with a previous melanoma diagnosis (*χ*^2^=7.04, *df*=1, *P*=0.008). In all, 50% of participants reported receiving a recommendation from a health professional to have a CSE and 59% had received a recommendation to perform SSE, although only 34% reported that the clinician had provided education or instruction on how to perform SSE. A greater proportion of affected (86%) than unaffected (48%) participants reported receiving a recommendation for SSE (*χ*^2^=15.45, *df*=1, *P*<0.001). Similarly, more affected (77%) than unaffected (39%) participants reported receiving a recommendation for CSE (*χ*^2^=14.16, *df*=1, *P*<0.001).

### Engagement in skin cancer screening behaviours

**Figure 1:**
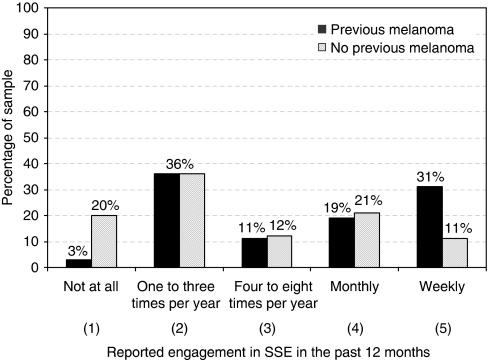
Reported frequency of SSE in the past 12 months, presented separately for those previously affected or unaffected by melanoma.

**Figure 2:**
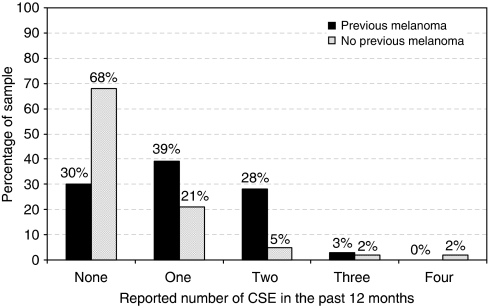
Reported frequency of CSEs in the past 12 months, presented separately for those previously affected or unaffected by melanoma.

Figures 1 and 2 illustrate annual frequency of SSE and CSE, respectively; data are presented separately for those previously affected or unaffected by melanoma. Overall, 21% of participants reported performing SSE once per month and 17% reported weekly performance of SSE. In contrast, 15% of participants had not performed SSE at all in the past 12 months and 36% ‘never’ or ‘rarely’ used a mirror or enlisted the help of another person when performing SSE. Those previously affected by melanoma reported significantly greater engagement in SSE over the past year (mean scale score *M*=3.39, s.d.=1.34) than unaffected (*M*=2.67, s.d.=1.31) participants (*Z*=2.71, *P*=0.007). Of those who reported not receiving a recommendation for SSE (*n*=49), 29% had not undertaken any form of SSE in the past 12 months. In terms of CSE behaviours, 43% of participants had engaged in CSE at least once in the past 12 months, with only 17% adhering to the recommended biannual uptake of CSE. The majority of the sample (57%) had no CSE in the past 12 months and 27% of participants never had a CSE. Affected participants (*M*=1.03 times, s.d.=0.85) reported significantly higher CSE uptake in the past 12 months than unaffected (*M*=0.48, s.d.=0.89) participants (*Z*=3.86, *P*=0.0005). Of those who reported not receiving a recommendation for CSE (*n*=59), 88% had no CSE in the past 12 months.

### Correlates of SSE behaviour

**Figure 3:**
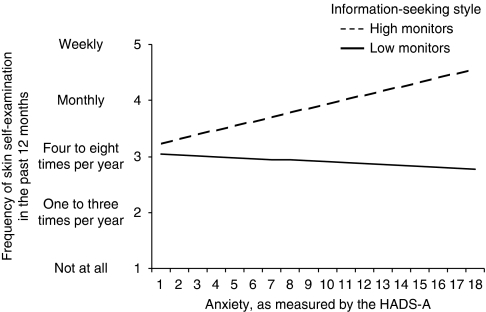
Mean annual frequency of SSE as a function of the interaction between information seeking style (low *vs* high monitoring) and anxiety, measured by the Hospital Anxiety and Depression Scale.

Demographic, clinical and psychological variables associated with SSE behaviour at the bivariate level are presented in Table 2. After allowing for the other predictors, doctor recommendation (*β*=1.77, 95% confidence interval (CI): 1.26–2.48, *P*=0.001), confidence in one's ability to perform SSE (*β*=1.44, 95% CI: 1.20–1.72, *P*<0.0001), positive beliefs about melanoma treatment (*β*=0.77, 95% CI: 0.65–0.91, *P*=0.002) and intention to perform SSE in the future (*β*=1.69, 95% CI: 1.45–1.99, *P*<0.0001) were independently associated with SSE uptake. Further, as illustrated by Figure 3, information-seeking style moderated the relationship between anxiety and SSE behaviour (*β*=1.02, 95% CI: 1.01–1.03, *P*=0.004). For ‘high monitors’, reported SSE was greater for those with higher generalised anxiety levels, compared with those with lower anxiety. In contrast, no difference in SSE behaviour was found for ‘low monitors’ across levels of anxiety. Overall, this model accounted for 59% of the variance in SSE behaviour.

### Correlates of CSE behaviour

Variables associated with CSE behaviour at the bivariate level are presented in Table 3. After allowing for education and the other predictor, doctor recommendation (*β*=2.21, 95% CI: 1.65–2.94, *P*<0.0001) and intention to undergo CSE in the future (*β*=1.19, 95% CI: 1.07–1.32, *P*=0.0008) were independently associated with CSE uptake. Overall, this model accounted for 40% of the variance in CSE uptake.

## Discussion

This study is among the first to investigate the frequency of self-reported skin cancer screening behaviours, as well as the correlates of these behaviours, in a sample of individuals at high risk of developing melanoma due to strong family history. Overall, in comparison with evidence-based clinical practice guidelines (Australian Cancer Network, 2008), we found that a substantial subset of individuals at increased risk of developing melanoma engage in suboptimal levels of skin cancer screening and surveillance. Over one-quarter of participants in this high-risk sample had never had a CSE, and only 17% reported adherence to the recommended biannual uptake of CSE. A similar pattern of results was found for SSE, with only 21% of the sample reporting monthly performance of SSE. Those without a personal history of melanoma reported significantly lower levels of screening, as well as lower levels of doctor–patient communication about melanoma and health-related behaviours. Given the genetic risk status of the families in this study, as well as the environmental risk associated with living in Australia, these findings are cause for concern.

Drawing comparisons between these findings and the screening behaviours of other high-risk melanoma samples is difficult, because of the paucity of published data. The low rate of monthly SSE observed in our sample is similar to that reported for individuals from *CDKN2A* mutation-positive families in the United States (17% Aspinwall *et al*, 2008), as well as melanoma survivors (15% Manne and Lessin, 2006) and individuals with multiple dysplastic nevi (10–20% Oliveria *et al*, 2004). Although it is possible that low levels of SSE may be associated with a greater reliance on regular CSE for early detection of skin cancer – with patients believing they are better off ‘leaving it up to the experts’ – this is highly unlikely in the present sample. Only 43% of participants had engaged in CSE in the past 12 months, a rate lower than the 52% observed by Aspinwall *et al* (2008) in their recent study of *CDKN2A* mutation-positive families. A *post hoc* analysis was conducted to investigate this possibility, and CSE and SSE uptake were found to be positively correlated (*r*_s_=0.22, *P*=0.02), indicating that an individual who conducted CSE was more – rather than less – likely to also conduct SSE. It is also notable that a sizeable proportion of the present sample reported weekly engagement in thorough SSE (i.e., over-screening; see Figure 1), a finding which is not uncommon among those at increased risk (Manne and Lessin, 2006; Aspinwall *et al*, 2008), but which is suggestive of a lack of understanding of the rationale for SSE. Weekly performance of SSE, reported by approximately one-third of those with a personal history of melanoma, may serve to diminish one's capacity to detect subtle but important skin changes. At the opposite end of the spectrum, 20% of participants without a personal history of melanoma reported no engagement in SSE over the past 12 months. Thus, across a range of high-risk melanoma populations from a variety of different geographical locations, there is growing evidence of inadequate uptake of skin cancer screening behaviours.

From a theoretical perspective, the study findings provide partial support for the Transactional Model of Stress and Coping. We found that the strongest predictor of both CSE and SSE behaviour was having received a recommendation from a health professional to perform screening. This finding is consistent with the broader cancer literature which shows that doctor recommendation is the single most important predictor of whether an individual has ever had a cancer screening test or has recently practiced screening (Rimer *et al*, 1991). This highlights the critical role that clinicians have in encouraging and supporting patients’ uptake of skin cancer-related health behaviours. It also suggests that clinician consultation may be a powerful yet relatively inexpensive vehicle for health behaviour change in this setting, with general practitioner or family physician consultations providing opportune moments for health behaviour promotion. For families with an inherited pattern of melanoma, genetic consultations may also provide a unique opportunity for tailored education and support with regard to skin cancer screening. Coupled with the finding that behavioural intentions also have a key role in determining uptake of CSE and SSE, this suggests that doctor–patient communication may help to increase screening intentions as well as behaviours in high-risk melanoma populations. The development of simple, evidence-based psycho-educational tools, such as a tailored discussion guide or a health behaviour ‘contract’, may serve to facilitate this communication process and elicit strong behavioural intentions among patients. In future work, researchers, clinicians and consumer representatives could partner to develop and evaluate the efficacy and cost-effectiveness of such tools in increasing skin cancer screening adherence. Given that only 50% of our sample reported receiving health professional advice about CSE, and only one-third reported receiving instructions on how to perform SSE, there appears to be considerable potential for improvement in this area.

In line with the Transactional Model, confidence in one's ability to perform SSE (i.e., self-efficacy) was also found to have an independent role in determining engagement in SSE. This reiterates the importance of not only educating patients about the need to perform SSE, but also providing individuals with the time and space to learn and practice the specific skills required for adequate SSE. In a previous study involving individuals with multiple dysplastic nevi, Oliveria *et al*, (2004) found that participation in a brief educational intervention featuring the use of digital photographs and nurse-delivered instruction on how to use the photographs led to substantial improvements in reported rates of SSE over a 4-month period. Education only, without the practical skills-based component, failed to produce an increase in SSE behaviour. Future studies could evaluate the efficacy of such interventions over a longer period, and in different populations (e.g., those with a strong family history of melanoma), to assess the longevity of improvements in SSE. As has been found with other health behaviour interventions (e.g., Myers *et al*, 2004), it may be necessary to supplement the initial training session with periodic prompts to action, as well as opportunities for ‘checking in’ to assess the adequacy and thoroughness of an individual's SSE technique. It is also imperative that future researchers monitor emotional responses to SSE-related education, as anxiety levels may increase as the focus on melanoma detection increases (for a review see Kasparian *et al*, 2009a).

We also found that information-seeking style moderated the relationship between anxiety and SSE behaviour. In particular, high monitors with higher levels of anxiety reported greater engagement in SSE than did high monitors with low anxiety. In contrast, there was no difference in SSE for low monitors across levels of anxiety. This finding is consistent with previous theoretical and empirical work, and supports the notion that, although anxiety drives high monitors to action, no amount of anxiety will galvanise those with a tendency to avoid risk-related information. It is possible, however, that higher anxiety in high monitors may lead to overscreening or an unhealthy focus on one's skin and moles. Thus, it may be important for clinicians to explore individuals’ beliefs about melanoma and SSE and to gently challenge any misconceptions that may arise.

Contrary to our initial hypothesis, perceived risk was not associated with screening uptake. In populations at an increased risk of melanoma, the role of perceived risk in determining screening behaviour is equivocal. Some studies have reported a positive association between perceived risk and SSE (Robinson *et al*, 2002), others have found only weak support for the association between SSE and perceived risk (Manne *et al*, 2004), and in other studies, perceived risk did not emerge as a predictor of SSE uptake (Geller *et al*, 2003). These mixed findings suggest that there may be some aspects of existing health behaviour models that do not adequately account for the results found in high-risk cancer populations, signalling the need to rethink conceptualisations of health behaviour in these populations.

### Recommendations for clinical practice and future research

It is hoped that these findings will contribute to the growing body of evidence indicating an urgent need to bolster health promotion and education efforts targeting populations at increased risk of melanoma. The results of this study provide some guide as to the key areas or ‘hot spots’ on which to focus attention when designing supportive care interventions for melanoma survivors and those at high risk of skin cancer. Clearly, it is imperative to engage clinicians (e.g., dermatologists, general practitioners, nurses, geneticists, genetic counsellors and psychologists) in the health behaviour change process, given that clinician recommendation was the strongest predictor of both CSE and SSE uptake. The development of programs and resources to improve skin cancer screening behaviours must include input and foster ownership from those in clinical practice. The same can be said for consumer representation. Without input from these key stakeholders, prevention efforts in this setting are unlikely to succeed. It is also critical that interventions address the role of psychological factors in health behaviour change. To this end, it is recommended that supportive care programs feature several components. First is psycho-education, which includes information on melanoma and melanoma risk management, education about the emotional, behavioural, physical and practical issues faced by individuals at increased risk of melanoma, exercises to assist in identifying and understanding negative core beliefs about skin cancer and screening, as well as tools to facilitate healthy coping strategies and open communication between patients and clinicians. Second, skills-based training is required to assist individuals in developing both confidence and capability in self-screening techniques. Third, sufficient time and space is needed for individuals to explore and express their feelings and concerns about melanoma with a caring professional who can listen attentively and try to understand.

Given the limited available data on the screening practices of those at high risk of melanoma, the findings of the present study make an important contribution to the literature. However, this study is not without its limitations. Owing to the cross-sectional study design, the data cannot elucidate the causal direction of associations. Because the findings are based on self-report, it is not possible to rule out the influence of socially desirable responding; however, given the low levels of adherence to clinical recommendations, the influence of this is likely to have been minimal. Further, the thoroughness of SSE was not directly assessed, suggesting some bias in the way this may have been interpreted by participants. Also, notification of the presence of a family-specific mutation in the *CDKN2A* gene is an event that would be expected to increase the self-perception of melanoma risk. In the present study, all participants were notified of the presence of a family-specific mutation in a single wave and no participants had undergone individual genetic testing in the clinical setting. Thus, it was not possible to examine the potential effect of time since notification on skin cancer screening behaviours. This is an important area for future research. Despite these limitations, identifying the characteristics of those most likely to adhere to screening recommendations (as well as the factors that may contribute to non-adherence) may enhance the effectiveness of supportive care programs and resources, as well as more widespread public health messages about the importance of skin cancer surveillance practices.

## Figures and Tables

**Table 1:** Mean scores for psychological variables, presented separately for those previously affected or unaffected by melanoma, as well as for the total sample

	**Affected (*n*=36)**	**Unaffected (*n*=84)**	**Total sample (*n*=120)**
**Variable**	**Mean (s.d.)**	**Mean (s.d.)**	**Mean (s.d.)**
Perceived risk	3.81 (0.66)	3.35 (0.74)	3.48 (0.74)
			
*Causal attributions*			
Perceived importance of sun exposure	4.32 (0.62)	4.14 (0.61)	4.19 (0.62)
Perceived importance of genetics	4.52 (0.52)	4.26 (0.69)	4.34 (0.65)
Perceived importance of uncontrollable factors	1.70 (0.99)	1.53 (0.67)	1.58 (0.77)
Self-efficacy for SSE	3.47 (1.06)	2.65 (0.91)	2.90 (1.02)
Perceived efficacy of CSE	1.33 (0.63)	1.44 (0.67)	1.41 (0.66)
Perceived efficacy of melanoma treatment	1.61 (0.84)	2.00 (0.99)	1.88 (0.96)
Intention to engage in SSE in future	3.78 (1.10)	3.50 (1.09)	3.58 (1.10)
Intention to engage in CSE in future	3.97 (1.16)	2.98 (1.41)	3.28 (1.41)
Melanoma-specific distress	10.78 (10.86)	5.49 (10.04)	7.08 (10.53)
General anxiety	4.89 (3.29)	5.68 (3.91)	5.44 (3.74)
General depression	2.94 (2.76)	3.55 (2.63)	3.37 (2.67)
Information-seeking style	8.14 (3.56)	8.45 (3.28)	8.36 (3.35)

Abbreviations: CSE=clinical skin examination; SSE=skin self-examination.

**Table 2:** Bivariate analysis of demographic, clinical and psychological variables associated with performance of skin self-examination in the past 12 months

	**Test statistics**			
**Variable**	**Mean SSE (s.d.)**	***Z***	***df***	***P***
*Demographic variables*				
*Sex*				
Male	2.95 (1.41)	0.49		0.62
Female	2.82 (1.31)			
*Marital status*				
Currently married	2.85 (1.26)	0.22		0.82
Not married	2.97 (1.62)			
*Education level*				
University degree	3.00 (1.29)	0.40		0.69
No university degree	2.87 (1.37)			
Age^a^		1.86	1	0.17
*Occupational environment*^b^				
Indoors	2.63 (1.27)	3.65	2	0.16
Outdoors	2.93 (1.27)			
Even time indoors and outdoors	3.22 (1.50)			
				
*Clinical variables*				
*Personal history of melanoma*				
Previous melanoma	3.39 (1.33)	2.71		**0.007**
No previous melanoma	2.67 (1.31)			
Number of FDRs and SDRs affected by melanoma^a^		5.66	1	**0.02**
Number of FDRs and SDRs deceased owing to melanoma^a^		0.026	1	0.87
*Objective CDKN2A mutation carrier risk*^b^				
100%	3.39 (1.34)	7.86	2	**0.02**
50%	2.75 (1.36)			
25% or less	2.53 (1.24)			
*Doctor recommendation for SSE*				
Yes	3.31 (1.31)	4.20		**<0.0001**
No	2.27 (1.19)			
*Doctor instruction on SSE*				
Yes	3.49 (1.23)	3.62		**<0.0001**
No	2.57 (1.32)			
				
*Psychological variables*		***r*_s_**		***P***
Perceived risk		0.15		0.10
*Causal attributions*				
Sun exposure		0.13		0.17
Genetics		0.05		0.59
Uncontrollable factors		0.02		0.82
Self-efficacy for SSE		0.52		**<0.0001**
Perceived efficacy of melanoma treatment		−0.25		**0.007**
Intention to engage in SSE in future		0.61		**<0.0001**
Melanoma-specific distress		0.25		**0.006**
General anxiety		0.09		0.34
General depression		0.06		0.53
*Information-seeking style*^c^		***Z***		***P***
High monitor	2.80 (1.38)	0.66		0.51
Low monitor	2.94 (1.31)			

Abbreviations: FDR=first-degree relatives; SDR=second-degree relatives; SSE=skin self-examination.

To interpret mean SSE scores, response options were: 1=‘not once’, 2=‘one to three times per year’, 3=‘four to eight times per year’, 4=‘once per month’ and 5=‘once per week’.

a Linear-by-linear association test.

b Kruskall–Wallis.

c Mann–Whitney *U*. Bold type is used to indicate statistical significance at the *P*<0.05 level.

**Table 3:** Bivariate analysis of demographic, clinical and psychological variables associated with frequency of clinical skin examination in the past 12 months

	**Test statistics**			
**Variable**	**Mean CSE (s.d.)**	***Z***	***df***	***P***
*Demographic variables*				
*Sex*				
Male	0.74 (1.00)	0.92		0.36
Female	0.56 (1.81)			
*Marital status*				
Currently married	0.68 (0.96)	0.26		0.80
Not married	0.56 (0.76)			
*Education level*				
University degree	1.00 (1.16)	1.20		0.23
No university degree	0.61 (0.87)			
Age^a^		0.54	1	0.46
Occupational environment^b^				
Indoors	0.69 (0.92)	4.24	2	0.12
Outdoors	0.36 (0.68)			
Even time indoors and outdoors	0.74 (0.86)			
				
*Clinical variables*				
*Personal history of melanoma*				
Previous diagnosis	1.03 (0.85)	3.86		**<0.0001**
No previous diagnosis	0.48 (0.89)			
Number of FDRs and SDRs affected by melanoma^a^		2.92	1	0.09
Number of FDRs and SDRs deceased owing to melanoma^a^		0.40	1	0.53
*Objective CDKN2A mutation carrier risk*^b^				
100%	1.03 (0.85)	16.07	2	**<0.0001**
50%	0.53 (0.86)			
25% or less	0.41 (0.95)			
*Doctor recommendation for CSE*				
Yes	1.14 (0.99)	6.69		**<0.0001**
No	0.14 (0.39)			
				
*Psychological variables*		***r*_s_**		***P***
Perceived risk		0.16		0.08
*Causal attributions*				
Sun exposure		0.11		0.22
Genetics		0.12		0.22
Uncontrollable factors		0.04		0.65
Perceived efficacy of CSE		−0.21		**0.02**
Perceived efficacy of melanoma treatment		−0.13		0.17
Behavioral intention to perform CSE		0.54		**<0.0001**
Melanoma-specific distress		0.16		0.08
General anxiety		−0.06		0.53
General depression		−0.09		0.33
*Information-seeking style*^c^		***Z***		***P***
High monitor	0.62 (0.84)	0.24		0.81
Low monitor	0.70 (0.99)			

Abbreviations: CSE=clinical skin examination; FDR=first-degree relatives; SDR=second-degree relatives.

a Linear-by-linear association test.

b Kruskall–Wallis.

c Mann–Whitney *U*. Bold type is used to indicate statistical significance at the *P*<0.05 level.
